# Identification of m6A/m5C/m1A-associated LncRNAs for prognostic assessment and immunotherapy in pancreatic cancer

**DOI:** 10.1038/s41598-023-30865-9

**Published:** 2023-03-04

**Authors:** Yuquan Huang, Wu Zhang, Qingxia Li, Zhe Wang, Xianghong Yang

**Affiliations:** 1 Department of Pathology, Shengjing Hospital of China Medical University, Shenyang, 110004 China; 2 North China University of Science and Technology, TangShan, 063210 China; 3 The Fourth Department of Oncology, Hebei General Hospital, Shijiazhuang, 050057 China

**Keywords:** Cancer, Immunology, Biomarkers, Oncology

## Abstract

Methylation of RNA plays an important role in cancer. Classical forms of such modifications include N6-methyladenine (m6A), 5-methylcytosine (m5C), and N1-methyladenine (m1A). Methylation-regulated long non-coding (lnc) RNAs are involved in various biological processes, such as tumor proliferation, apoptosis, immune escape, invasion, and metastasis. Therefore, we performed an analysis of transcriptomic and clinical data of pancreatic cancer samples in The Cancer Genome Atlas (TCGA). Using the co-expression method, we summarized 44 m6A/m5C/m1A-related genes and obtained 218 methylation-associated lncRNAs. Next, with COX regression, we screened 39 lncRNAs that are strongly associated with prognosis and found that their expression differed significantly between normal tissues and pancreatic cancer samples (P < 0.001). We then used the least absolute shrinkage and selection operator (LASSO) to construct a risk model comprising seven lncRNAs. In validation set, the nomogram generated by combining clinical characteristics accurately predicted the survival probability of pancreatic cancer patients at 1, 2, and 3 years after diagnosis (AUC = 0.652, 0.686, and 0.740, respectively). Tumor microenvironment analysis showed that the high-risk group had significantly more resting memory CD4 T cells, M0 macrophages, and activated dendritic cells and fewer naïve B cells, plasma cells, and CD8 T cells than the low-risk group (both P < 0.05). Most immune-checkpoint genes were significantly different between the high- and low-risk groups (P < 0.05). The Tumor Immune Dysfunction and Exclusion score showed that high-risk patients benefited more from treatment with immune checkpoint inhibitors (P < 0.001). Overall survival was also lower in high-risk patients with more tumor mutations than in low-risk patients with fewer mutations (P < 0.001). Finally, we explored the sensitivity of the high- and low-risk groups to seven candidate drugs. Our findings indicated that m6A/m5C/m1A-associated lncRNAs are potentially useful biomarkers for the early diagnosis and estimating the prognosis of, and ascertaining the responses to immunotherapy in, patients with pancreatic cancer.

## Introduction

Pancreatic ductal adenocarcinoma is among the most lethal malignancies of the digestive system^1^ and is expected to become the second leading cause of cancer-related deaths in 20–30 years^2^. Surgical resection is the only treatment option for patients with lesions localized to the pancreas^3^. However, owing to the lack of early symptoms and specific markers, by the time most patients are diagnosed, they already have advanced pancreatic cancer, and the treatment window has passed^4^. Tumor heterogeneity and unique immune-evasion mechanisms also increase the difficulty of treating pancreatic cancer^5^. Molecular biomarkers with diagnostic, predictive, and therapeutic value are thus urgently needed.

Epigenetics plays an important role in tumorigenesis, of which the most well-known processes include DNA methylation, RNA modifications, and chromatin remodeling^6^. In particular, chemical modifications of RNA, especially, methylation, are critical to gene and protein expression^7^. Methylation of RNA (m6A, m1A, and m5C) is an increasingly popular research topic. The methylation reaction is catalyzed by the methyltransferase complex comprising a methyltransferase (“writer”), a demethylase (“eraser”), and a binding protein (“reader”)^8^. The m6A modification has been associated with multiple cancers, including kidney, breast, lung, and ovarian cancers^9–13^; the m1A modification is highly expressed in bladder uroepithelial carcinoma and is a candidate biomarker for the early diagnosis of bladder cancer^14^. Meanwhile, the m5C modification plays an important role in the proliferation of pancreatic cancer cells^15^. Furthermore, the m5C-mediated upregulation of long non-coding RNAs (lncRNAs; > 200 nucleotides in length) was significantly enhanced in esophageal squamous cell carcinoma and was associated with tumor migration, invasion, and drug resistance^16^.

Several studies have revealed that lncRNAs play a role in epigenetics, tumor immunity, immune-cell activation and distribution, and immune escape of tumor cells^17–19^. These varied functions have led to the suggestion that lncRNAs can be used as specific biomarkers with diagnostic and prognostic value and can predict treatment response^20^. For example, urinary levels of PCA3 aid the diagnosis of prostate cancer^21^, and HOTAIR can predict the response to cisplatin therapy in patients with ovarian cancer^22^. However, limited data regarding the role of methylation-mediated lncRNAs in pancreatic cancer are available. To improve the diagnosis, prognosis, and therapy outcomes for pancreatic cancer, the relationship between RNA methylation and lncRNAs requires further exploration.

In the present study, we combined the transcriptomic and clinical data of 177 pancreatic cancer patients to construct m6A/m5C/m1A-related prognostic models. Our findings clarify the potential of related lncRNAs for use as biomarkers for pancreatic cancer.

## Materials and methods

### Collection and processing of pancreatic cancer samples

Transcriptomic data corresponding to pancreatic cancer samples—from TCGA—and normal pancreatic tissues—from GTEx—were obtained through UCSC Xena (https://xenabrowser.net/datapages/). Clinical data related to the pancreatic cancer samples (mutations and immunophenotyping) were collected from TCGA (https://cancergenome.nih.gov/). Samples with missing overall survival (OS) data were excluded^23^. This study included 171 normal pancreatic tissues and 177 pancreatic cancer samples that were randomly divided into training and validation sets, with a ratio of 5:5.

### Identification of m6A/m5C/m1A-associated lncRNAs with prognostic value

Expression data of 44 m6A/m5C/m1A-related genes and 3752 lncRNAs were obtained from TCGA. Using Spearman’s correlations (ρ > 0.5 and P < 0.001), 218 lncRNAs were found to be co-expressed with m6A/m5C/m1A genes. Univariate COX regression analysis identified 39 lncRNAs with prognostic value based on survival data for each sample, and P < 0.001 was considered statistically significant.

### Clustering and analysis of pancreatic cancer samples based on the expression of m6A/m5C/m1A-associated lncRNAs

To elucidate lncRNA function in pancreatic cancer, tumor samples were classified into subtypes based on 39 prognostic lncRNAs using R package “ConsensusClusterPlus”^24^. Principal components analysis (PCA) was used to determine if the samples could be visually distinguished. Next, survival analysis for the different subtypes was performed using the R packages “survival” and “survminer.” Clinical data were also included to determine whether the observed subtypes differed clinically. GSEA version 4.2.3 was used to perform gene enrichment analysis on the tumor samples; gene sets were considered significant at P < 0.05.

CIBERSORT is an immune-cell-expression matrix deconvolution tool based on linear support vector regression. It detects marker gene expression to quantify the proportion of infiltrating immune cells. Based on the expression profiling data, CIBERSORT can analyze the abundance of 22 tumor-infiltrating immune cell (TIIC) isoforms from complex tissues^25^. Here, the immune infiltration fraction per tumor sample was calculated with CIBERSORT. The R package “limma” was used to observe variations in immune cell infiltration among the different subtypes^26^. Finally, the “ESTIMATE” R package—that uses gene expression profiles—was used to predict stromal and immune cell scores; this package was also used to calculate the number of both cell types to analyze the purity of pancreatic cancer samples^27^. The expression of *KRAS* was also explored across different subtypes.

### Construction and validation of risk prognostic model

TCGA pancreatic-cancer samples were randomly divided into training and validation sets. Using the R package “glmnet,” LASSO regression was performed. This analysis is a high-dimensional exponential regression method for constructing risk prediction models. Here, LASSO was used to screen m6A/m5C/m1A-associated prognostic lncRNAs and accordingly penalize the contraction of regression coefficients^28,29^. A risk model was built with seven lncRNAs in the training set and was validated on the full dataset and test set. Clinical parameters did not differ significantly between the two datasets (P > 0.05) (Supplementary Table 1). Correlation coefficients of the seven lncRNAs were determined, and the risk score of each tumor sample was calculated using the following formula: ${\text{Risk}}\;{\text{score}} = {\text{x}}_{{{\text{i}} = 1}}^{{\text{n}}} \;{\text{Coefi}} \times {\text{x}}^{{\text{i}}}$, where Coefi refers to coefficients, and x^i^ refers to the FPKM value of each m6A/m5C/m1A-related lncRNA. Univariate and multifactorial Cox regression analyses combined with clinical parameters (age, sex, grading, and staging) were used to assess whether the risk scores in the training and validation sets are useful as independent prognostic indicators. In addition, PCA was used to determine whether the samples could be separated into high- and low-risk groups based on risk scores. Model accuracy was assessed using ROC curves and the C-index.

### Nomogram construction

A nomogram was constructed with R package “rms” to predict the OS of pancreatic cancer patients at 1, 2, and 3 years post-diagnosis based on the risk scores and clinical characteristics (age, sex, grade, and stage). Agreement between the actual data and model predictions was assessed using a calibration curve.

### Exploring immune landscape and immunotherapeutic response in the prognostic model

The “limma” package was used to analyze the differences in immune cells and immune checkpoints between high- and low-risk groups. Potential immune-checkpoint genes were determined using a literature search. Between-group differences in tumor immune-microenvironment scores were also analyzed. Spearman’s correlations were calculated between risk scores and immune cells. Additionally, ssGSEA was used to explore the differences between the high- and low-risk groups in 29 immune-cell subsets and immune-related functional pathways. Finally, we obtained the TIDE (Tumor Immune Dysfunction and Exclusion, http://tide.dfci.harvard.edu/) scores of pancreatic cancer patients in TCGA dataset and inferred patient response to immune-checkpoint inhibition therapy^30^.

### Immune infiltration and prognostic analysis of lncRNAs

Samples in the model were divided into high- and low-lncRNA-expression groups; each lncRNA’s survival curve was calculated using the “survival” package in R. Based on published categories, tumor samples in TCGA were classified into six immune subtypes, i.e., wound healing (immune C1), IFN-gamma dominant (immune C2), inflammatory (immune C3), lymphocyte depleted (immune C4), immunologically quiet (immune C5), and TGF-beta dominant (immune C6)^31^. We separately explored the expression differences in the seven lncRNAs across immune subtypes in the risk model.

### Prognostic characteristics of tumor mutation burden

Mutation data were evaluated using the R package “maftools.” Based on the tumor mutation burden (TMB) and patient survival data, samples were divided into high-TMB and low-TMB groups. Survival analysis was performed using combined risk scores of patients in high- and low-TMB groups. Next, TMB differences between high and low-risk groups were compared, and correlations between risk score and TMB were calculated. Finally, a prognostic analysis was performed on wild-type and mutant KRAS genes in pancreatic cancer samples.

### Drug sensitivity analysis

Drug sensitivity of high- and low-risk pancreatic cancer samples was predicted using the R package “pRRophetic” based on clinical results from the Cancer Genome Project (CGP)^32^. Correlations between risk scores and drug sensitivity were also calculated.

## Results

### Identification of m6A/m5C/m1A-related lncRNAs in pancreatic cancer samples

**Figure 1:**
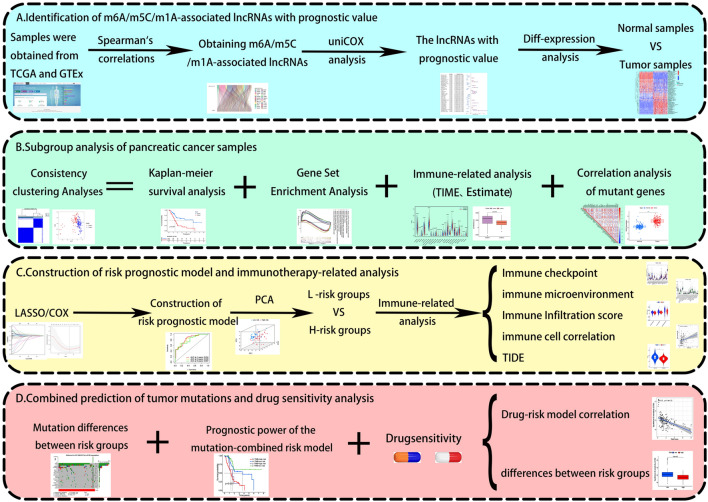
Study flow chart.

**Figure 2:**
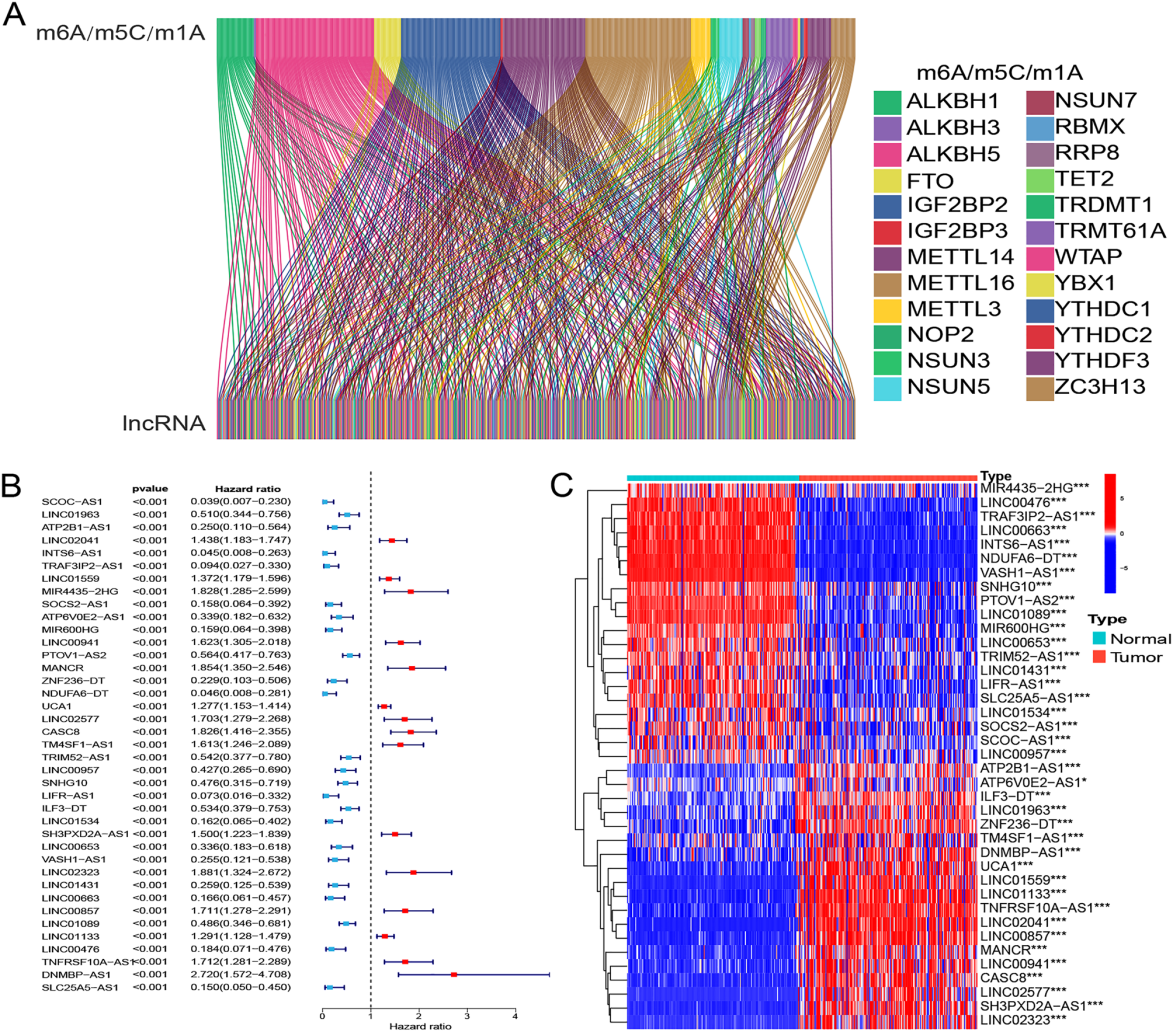
Filtering long non-coding (lnc)RNAs with prognostic potential for pancreatic cancer from a larger pool of methylation-associated lncRNAs.*, **, ***, represent P < 0.05, P < 0.01 and P < 0.001. (**A**) Sankey diagram of co-expression between m6A/m5C/m1A methylation types and lncRNAs. (**B**) Forest plot of 39 prognosis-related lncRNAs. (**C**) Heatmap of prognosis-related lncRNAs in pancreatic cancer samples and normal pancreatic tissue samples.

Study procedures are summarized in Fig. 1. Our literature search yielded 44 m6A/m5C/m1A-related genes (Table 1). The expression matrix of these 44 genes was extracted from 177 pancreatic cancer samples. Using Spearman’s correlations, we identified 218 methylation-related lncRNAs co-expressed with the 44 genes in pancreatic cancer samples (Fig. 2A). In combination with clinical data, we filtered prognosis-related lncRNAs (univariate Cox regression, P < 0.001) and identified 39 prognosis-associated lncRNAs (Fig. 2B). We further assessed the expression of the selected lncRNAs in pancreatic cancer samples and normal pancreatic tissues. Finally, we used a heat map to illustrate the significant difference in lncRNA expression between pancreatic cancer samples and normal pancreatic tissues, suggesting that methylation-related lncRNAs play an important role in pancreatic cancer (Fig. 2C).

**Table 1:** Methylation (m6A/m5C/m1A)-related genes.

RNA methylation	Writers	Readers	Erasers
m6A	METTL3, METTL14, METTL16, WTAP, RBM15, RBM15B, RBMY1A1, ZC3H13	YTHDC1, YTHDC2, YTHDF1, YTHDF2, YTHDF3, IGF2BP1, IGF2BP2, IGF2BP3, HNRNPA2B1, HNRNPC, RBMX, FMR1, LRPPRC	ALKBH5, FTO
m5C	NOP2, NSUN2, NSUN3, NSUN4, NSUN5, NSUN7, DNMT1, TRDMT1, DNMT3A, DNMT3B	ALYREF	TET2, YBX1
m1A	TRMT6, TRMT61A, TRMT61B, TRMT10C, BMT2, RRP8	YTHDC1, YTHDF1, YTHDF2	ALKBH1, ALKBH3

### Clustering of pancreatic cancer samples based the expression of m6A/m5C/m1A-related lncRNAs

**Figure 3:**
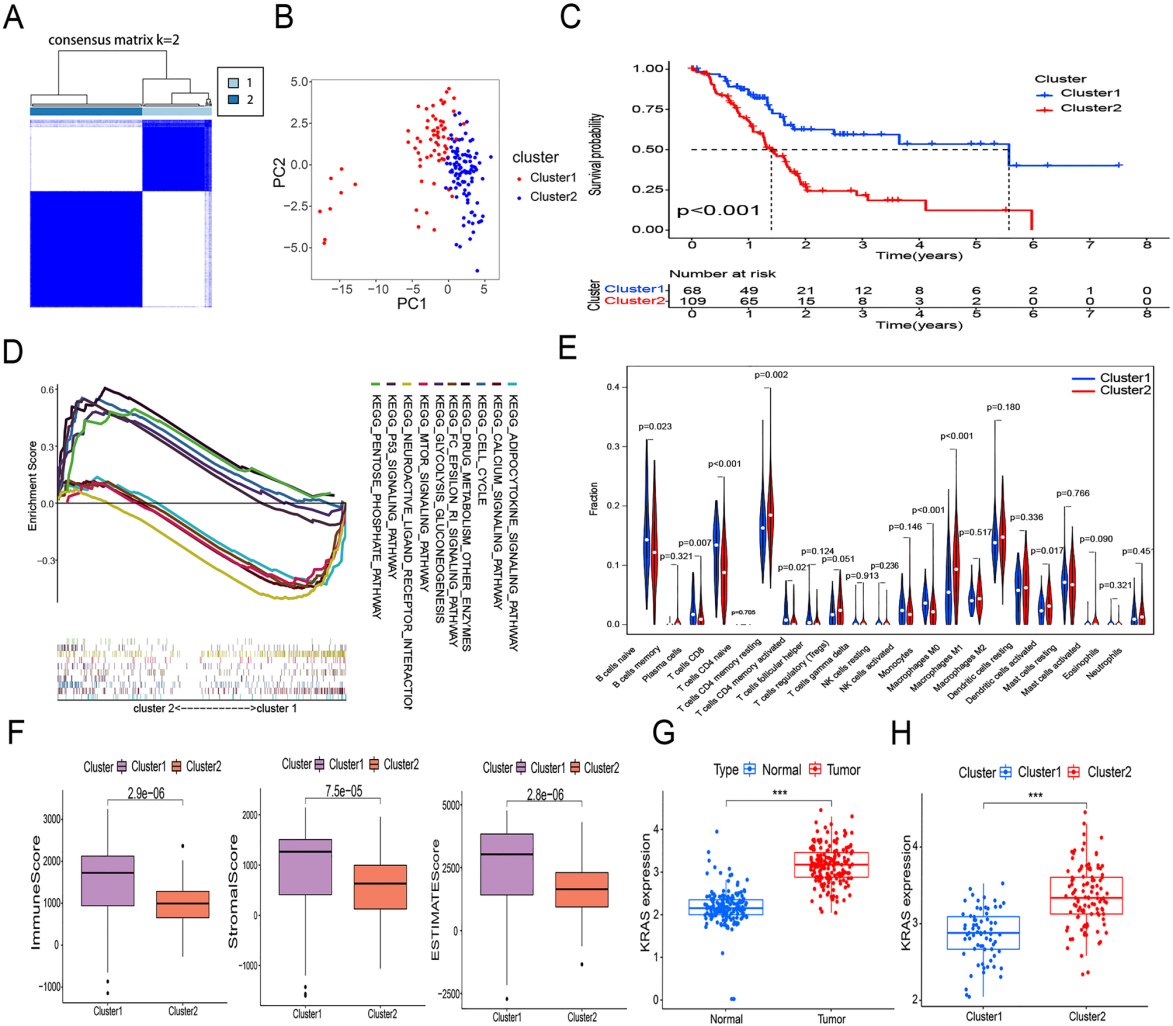
Prognosis and immune status of pancreatic cancer samples after clustering. *, **, ***, represent P < 0.05, P < 0.01 and P < 0.001. (**A**) Consistent clustering plot of pancreatic cancer samples. (**B**) Principal components analysis divided pancreatic cancer samples into Cluster1 and Cluster2. (**C**) Kaplan–Meier survival analysis for the two clusters (P < 0.001). (**D**) GSEA of Cluster1 and Cluster2. (**E**) Violin plot depicting the differential analysis of immune cell expression in the two clusters. (**F**) Boxplot of immune infiltration scores for Cluster1 and Cluster2, showing a significant between-group difference. (**G**, **H**) Boxplots of differences in KRAS genes between normal and pancreatic cancer samples, as well as between the two clusters.

Unsupervised cluster analysis classified pancreatic cancer samples into two categories (optimal K value = 2) (Fig. 3A); this division was supported by PCA (Fig. 3B). The Kaplan–Meier survival curve showed that significantly better prognosis was associated with Cluster1 samples than with Cluster2 samples (P < 0.001) (Fig. 3C). However, the two clusters did not differ significantly with respect to clinical parameters (Supplementary Fig. S1). Next, GSEA showed that Cluster2 pathways were mainly enriched in DRUG_METABOLISM_OTHER_ENZYMES, CELL_CYCLE, and CALCIUM_SIGNALING_PATHWAY terms. Cluster1 pathways were mainly enriched in MOTOR_SIGNALING_PATHWAY, NEUROACTIVE_LIGAND_RECEPTOR_INTERACTION, P53_SIGNALING_PATHWAY, and FC_EPSILON_RI_SIGNALING_PATHWAY terms (Fig. 3D). Immune microenvironment analysis suggested that CD8 T cells, activated memory CD4 T cells, plasma cells, monocytes, and naïve B cells were significantly more enriched in Cluster1 than in Cluster2, whereas resting memory CD4 T cells, M0 macrophages, and activated dendritic cells were more enriched in Cluster2 than in Cluster1 (P < 0.05, Fig. 3E). Cluster1 also had higher Immune, Stromal, and ESTIMATE scores than Cluster2 (P < 0.001, Fig. 3F). *KRAS* expression was significantly higher in Cluster2 than in Cluster1 (P < 0.001, Fig. 3G) and in pancreatic cancer samples than in normal samples (P < 0.001, Fig. 3H). The correlation heat map indicated that *KRAS* was correlated with prognostic lncRNAs (Supplementary Fig. S2).

### Risk model of m6A/m5C/ m1A-related lncRNAs

**Figure 4:**
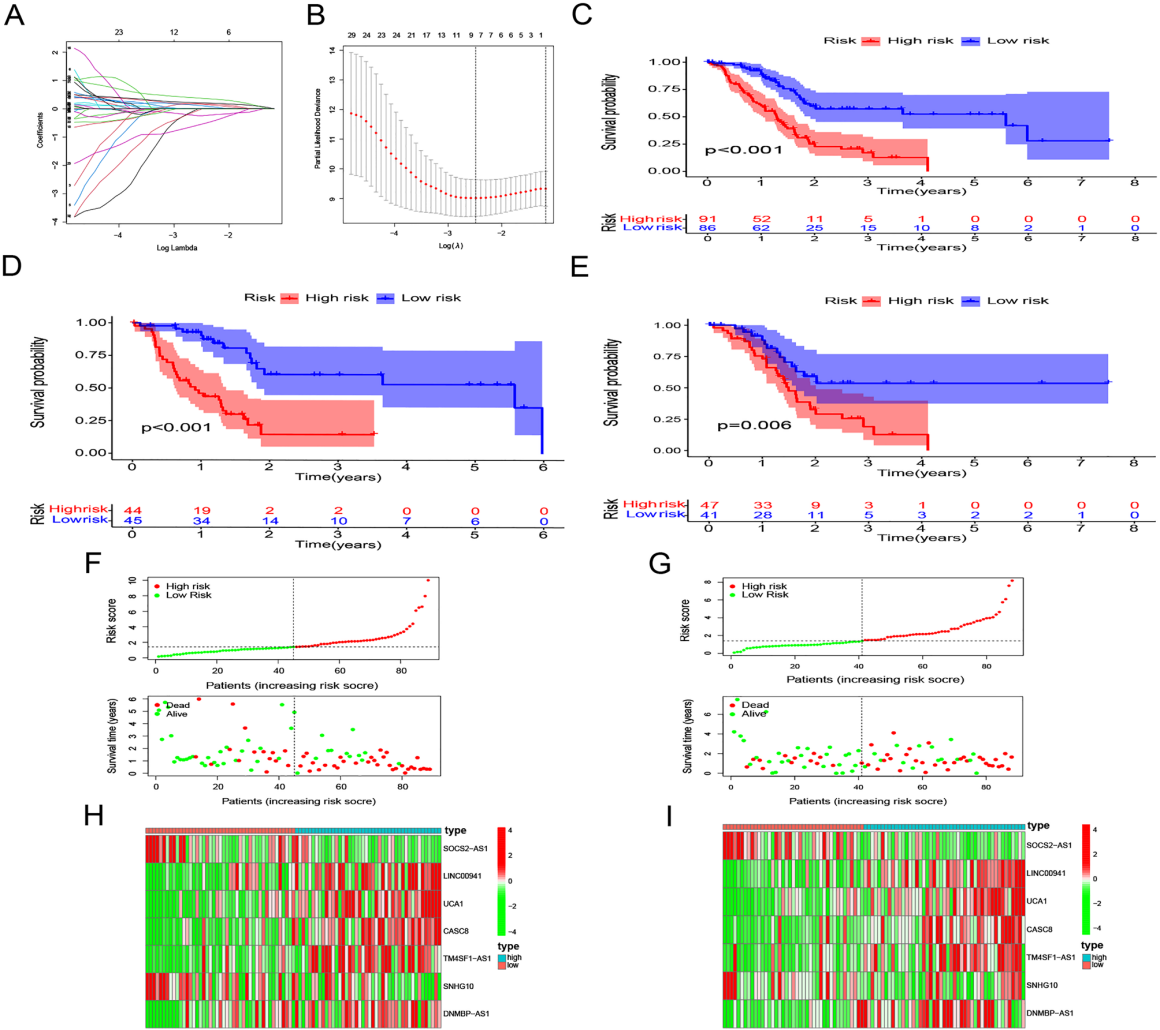
Models of m6A/m5C/m1A-related lncRNAs as pancreatic cancer markers. (**A**, **B**) Prognostic models for m6A/m5C/m1A-related lncRNAs were constructed using least absolute shrinkage and selection operator regression. (**C**–**E**) Kaplan–Meier survival curves between the high- and low-risk groups in the complete dataset, training set, and validation set. (**F**, **G**) Plots of risk score and survival status distribution in the training and validation sets. (**H**, **I**) Heatmaps of the seven lncRNAs in the training and validation sets.

To construct a prognostic risk model, of the initial 39 prognostic lncRNAs, we selected the following using the LASSO regression method: SOCS2-AS1, LINC00941, UCA1, CASC8, TM4SF1-AS1, SNHG10, and DNMBP-AS1 (Fig. 4A,B; see Table 2 for correlations). After using the median risk scores to classify pancreatic cancer samples into high- and low-risk groups, we found that high-risk patients had a significantly lower OS than low-risk patients (P < 0.001, Fig. 4C). The results were validated in the training (P < 0.003, Fig. 4D) and validation sets (P = 0.006, Fig. 4E). For both datasets, the percentage of deaths increased with increasing risk scores (Fig. 4F,G). Five lncRNAs (LINC00941, UCA1, CASC8, TM4SF1-AS1, and DNMBP-AS1) were more strongly expressed in the high-risk group than in the low-risk group, suggesting that they are the potential risk factors for pancreatic cancer. Moreover, as the expression of both SNHF10 and SOCS2-AS1 was lower in the high-risk group than in the low-risk group, they may serve as protective factors (Fig. 4H,I).

**Table 2:** Correlation coefficients of model lncRNAs.

Gene	SOCS2-AS1	LINC00941	UCA1	CASC8	TM4SF1-AS1	SNHG10	DNMBP-AS1
Coef	− 0.247837634189719	0.159718556194045	0.208243799404174	0.369953214750176	0.106004512577679	− 0.536305042722021	0.177106805600572

### Predictive value of the risk model

**Figure 5:**
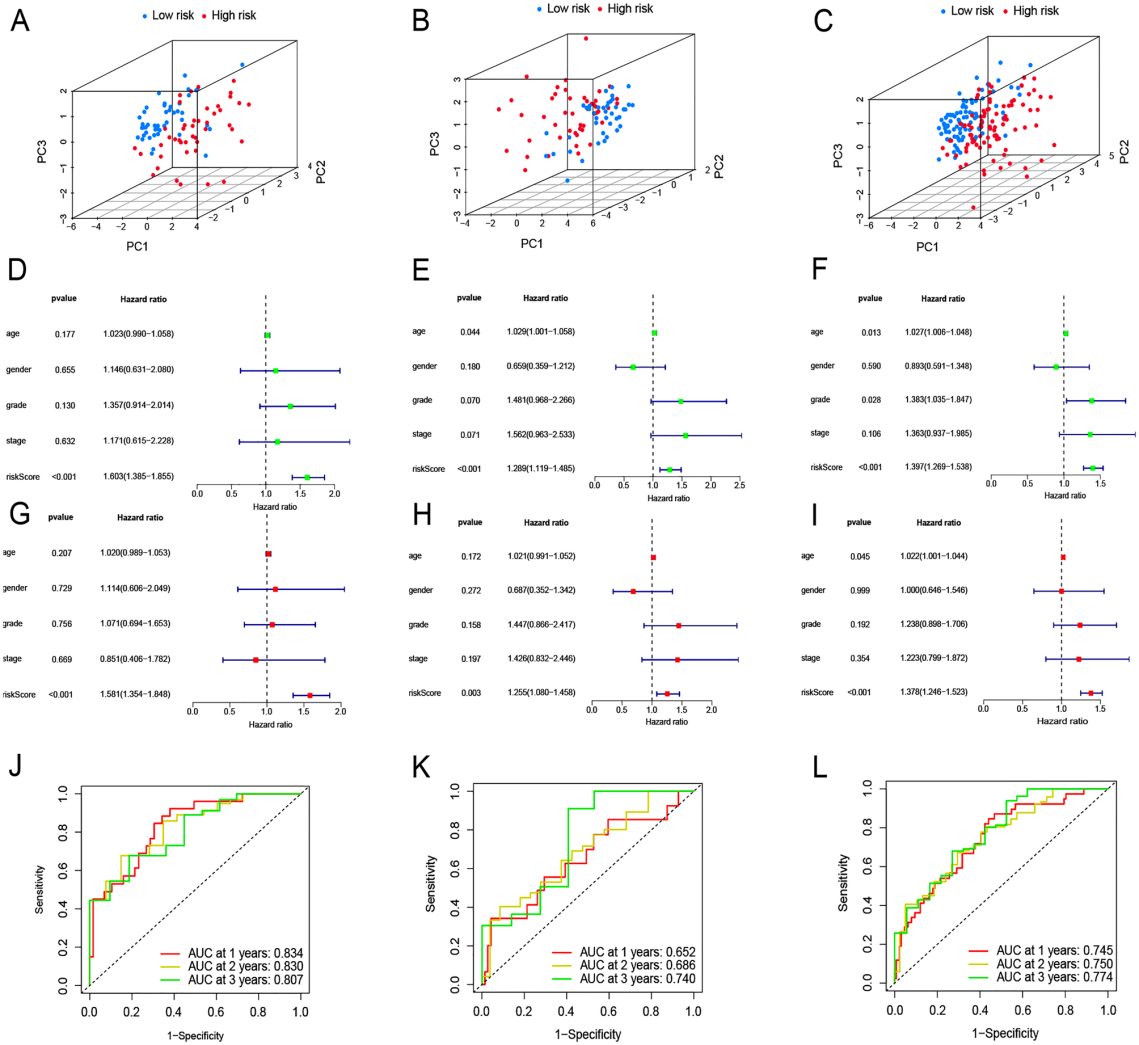
Evaluation of prognostic model efficacy. (**A**–**C**) Three-dimensional principal components analysis (3dPCA) of the high- and low-risk groups in the training set, validation set, and full dataset. (**D**–**F**) Univariate regression of the prognostic model in the training set, validation set, and full dataset. (**G**–**I**) Multifactorial regression of the prognostic model the training set, validation set, and full dataset. (**J**–**L**) ROC curves for 1 year, 2 years, and 3 years post-diagnosis the training set, validation set, and full dataset.

Three-dimensional PCA of the training, validation, and whole-sample sets demonstrated that patients were appropriately classified into low- and high-risk groups based on risk scores (Fig. 5A–C). Similarly, univariate COX regression on the three datasets indicated that the risk score is an independent prognostic indicator and has prognostic value compared to traditional clinical parameters (Fig. 5D–F); multifactor COX regression supported this conclusion (Fig. 5G–I). In the training set, the ROC curve values for 1, 2, and 3 years post-diagnosis were 0.834, 0.830, and 0.807, respectively (Fig. 5J). In the validation set, the corresponding ROC curve values were 0.652, 0.686, and 0.740, respectively (Fig. 5K). In the complete dataset, the ROC curve values were 0.745, 0.750, and 0.774 (Fig. 5L). For all three post-diagnosis years, these ROC curve values were better than traditional clinical parameters, such as age (AUC = 0.540), sex (AUC = 0.565), grade (AUC = 0.598), and stage (AUC = 0.483) (Supplementary Fig. S3A). The risk score also had a significantly better conformance index than the four clinical parameters (Supplementary Fig. S3B).

**Figure 6:**
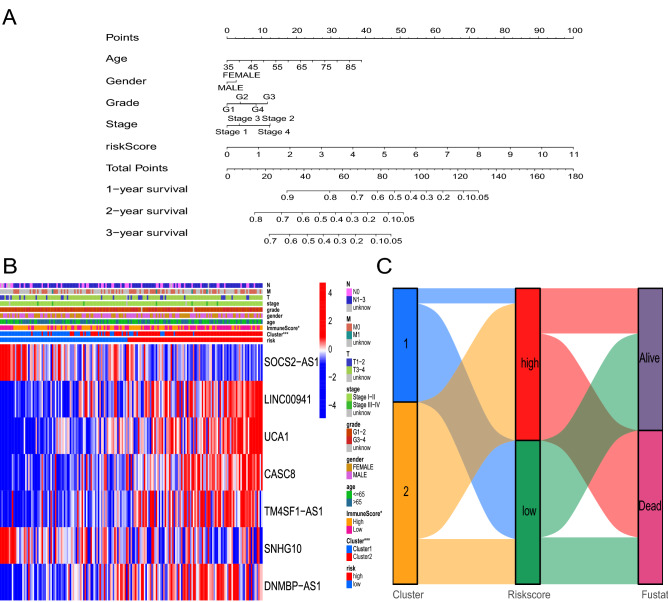
Nomogram construction and evaluation. (**A**) Nomogram for predicting the prognosis of patients with pancreatic cancer. (**B**) Heatmap showing the differential expression of the model lncRNAs. (**C**) Sankey diagram of cluster, risk score, and survival status.

To better predict the OS of pancreatic cancer patients, we constructed a nomogram based on the risk score combined with clinical parameters (age, sex, grade, and stage) (Fig. 6A). In addition, we used calibration curves to compare the actual and predicted survival probabilities at 1, 2, and 3 years. The results indicated that the two sets of probabilities were consistent over 3 years (Supplementary Fig. S4A), indicating that the generated nomogram was reliable. Next, we performed survival analysis within different age, sex, TNM stage, and tumor grade for the high- and low-risk groups. Patients with T1-2, T3-T4, N0, N1-3, M0, stage I-II, G1-G2, and G3-4 in the high-risk group had a worse prognosis than patients with similar parameters in the low-risk group (P < 0.05, Supplementary Fig. S4B), supporting the utility of our validating model. A heatmap revealed significant differences in the cluster and immune scores of the seven lncRNAs between the high- and low-risk groups (P < 0.05, Fig. 6B). Finally, the Sankey diagram showed that most Cluster2 samples were in the high-risk group and had poorer prognosis, confirming the results of previous survival analysis (Fig. 6C).

### Prognostic model of immune landscape and immunotherapeutic response

**Figure 7:**
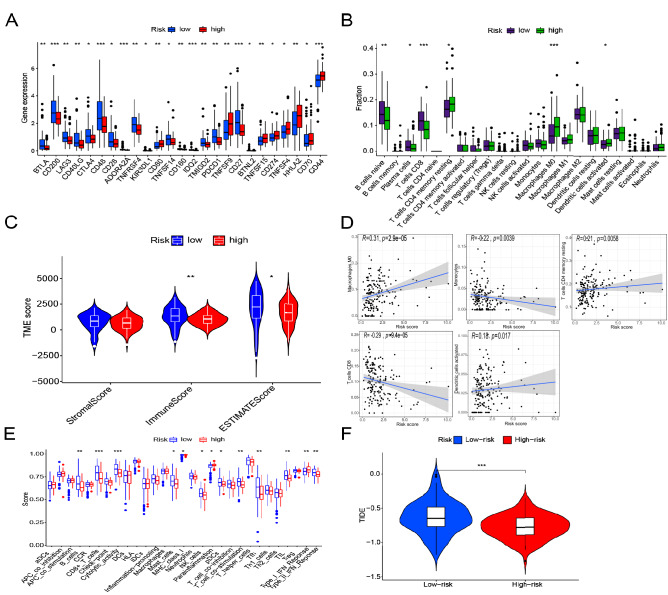
Immune landscape of the prognostic model. *, **, ***, represent P < 0.05, P < 0.01 and P < 0.001. (**A**) Boxplot showing the differential expression of immune checkpoint genes between the high- and low-risk groups. (**B**) Boxplot showing the differential expression of immune cells between the high- and low-risk groups. (**C**) Violin plot showing differences in the immune infiltration scores between groups. (**D**) Correlation of immune cells and risk score. (**E**) Boxplot showing differential expression of immune cell subpopulations and immune cell-related functions in high- and low-risk groups. (**F**) Violin plot of Tumor Immune Dysfunction and Rejection (TIDE) scores in the high- and low-risk groups.

The risk-prognosis model revealed 25 immune-checkpoint genes that were significantly different between the high- and low-risk groups (P < 0.05) (Fig. 7A). Specifically, *BTLA*, *CD200*, *LAG3*, *CD40LG*, *CTLA4*, *CD48*, *CD28*, *TNFRSF4*, *TNFSF14*, *TMIGD2*, *PDCD1*, and *CD27* were upregulated in the low-risk group, whereas *CD80*, *TNFSF9*, *TNFSF15*, *CD274*, *TNFSF4*, *HHLA2*, *CD70*, and *CD44* were upregulated in the high-risk group. Tumor immune-microenvironment analysis showed that the number of naïve B cells, plasma cells, and CD8 T cells was significantly higher in the low-risk group than in the high-risk group, whereas that of resting memory CD4 T cells, M0 macrophages, and activated dendritic cells were significantly higher in the high-risk group (P < 0.05, Fig. 7B). Moreover, the high-risk group had lower Immune and ESTIMATE scores (P < 0.05, Fig. 7C). Correlation analysis showed that activated dendritic cells, M0 macrophages, and resting memory CD4 T cells were positively correlated with risk scores, whereas CD8 T cells and monocytes were negatively correlated (P < 0.05, Fig. 7D). ssGSEA on immune cell subsets and immune cell-related functions revealed that B cells, CD8+ T cells, cytolytic activity, mast cells, NK cells, pDCs, co-stimulation T cells, Tfh, TIL, and type II IFN response were significantly higher in the low-risk group than in the high-risk group. However, type I IFN response was higher in the high-risk group than in the low-risk group (P < 0.05, Fig. 7E). Finally, the low-risk group had higher TIDE scores, indicating that high-risk patients had better outcomes with immunotherapy (P < 0.001, Fig. 7F).

### Prognostic analysis and immunophenotyping of model lncRNAs

**Figure 8:**
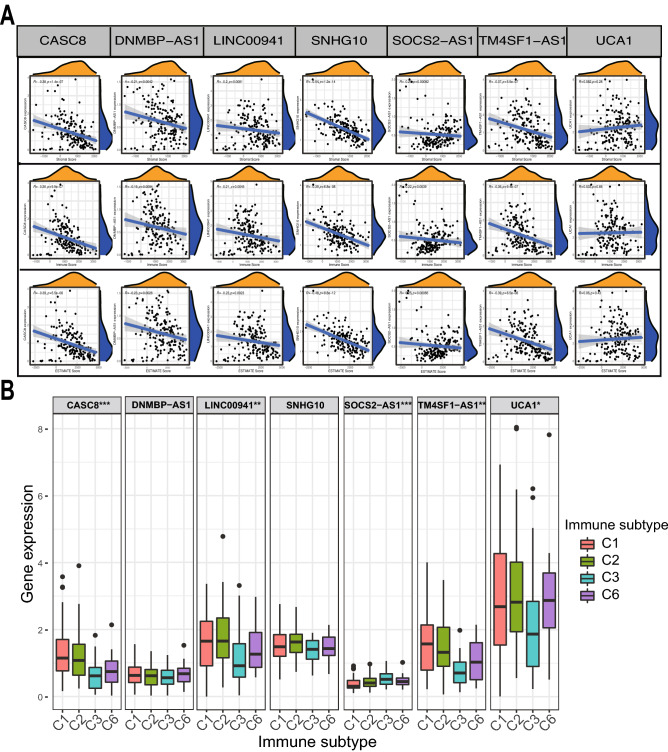
Correlation analysis of individual lncRNA expression and immune score in the model. *, **, ***, represent P < 0.05, P < 0.01 and P < 0.001. (**A**) Correlations between lncRNA expression and immune infiltration score. (**B**) Relationship between lncRNA expression and immune subtypes.

Survival analysis of pancreatic cancer samples showed that low expression of UCA1, TM4SF1-AS1, LINC00941, DNMBP-AS1, and CASC8 improved OS more than high expression, whereas high expression of SNHG10 and SOCS2-AS1 improved OS more than low expression (P < 0.001, Supplementary Fig. S5A). CASC8, DNMBP-AS1, LINC00941, TM4SF1-AS1, and UCA1 expression was positively correlated with risk scores, indicating that they are risk factors for pancreatic cancer; in contrast, SNHG10 and SOCS2-AS1 were negatively correlated, thus serving as protective factors for pancreatic cancer. These results confirmed our earlier results (P < 0.001, Supplementary Fig. S5B). Next, the expression of TM4SF1-AS1, LINC00941, DNMBP-AS1, CASC8, SNHG10, and SOCS2-AS1 was negatively correlated with the stromal, immune, and total scores (P < 0.05, Fig. 8A). Furthermore, immuno-infiltration analysis revealed that SOCAS2-AS1 was highly expressed in C3 and C6 but lowly expressed in C1 and C2 (P < 0.001); TM4SF1-AS1, CASC8, and LINC00941 were highly expressed in C1 and C2 but lowly expressed in C3 and C6 (P < 0.05); and UCA1 was highly expressed in C1, C2, and C6, but lowly expressed in C3 (Fig. 8B).

### Prognostic model of TMB and drug sensitivity

**Figure 9:**
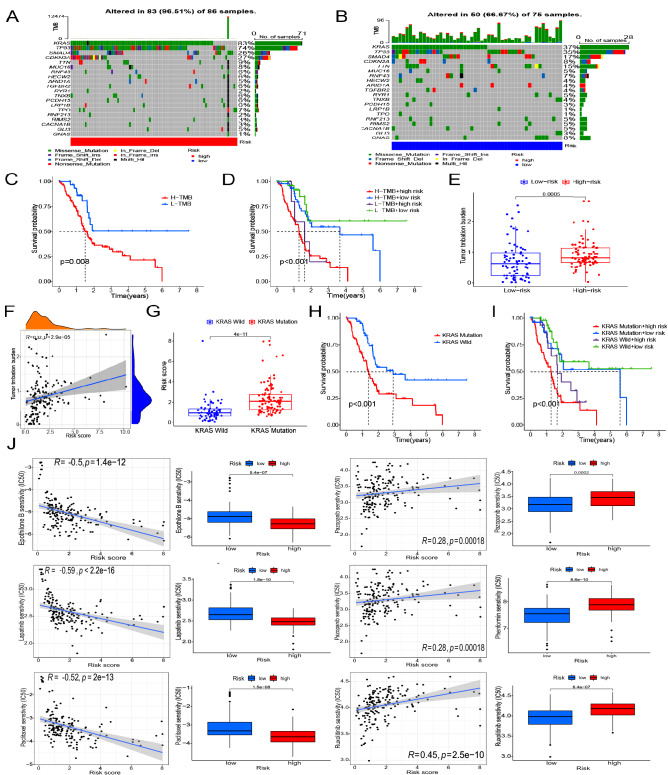
Tumor mutation burden (TMB), KRAS gene mutation, and drug sensitivity analysis. (**A**, **B**) Mutation waterfall plots for high- and low-risk groups. (**C**) Kaplan–Meier survival analysis between high and low TMB. (**D**) Kaplan–Meier survival analysis of different TMB levels combined with the risk score. (**E**) Analysis of TMB differences between high- and low-risk groups. (**F**) Correlation between risk score and TMB. (**G**) Boxplot showing differences in risk score between wild-type and mutant KRAS. (**H**) Kaplan–Meier survival analysis of wild-type and mutant *KRAS*. (**I**) Kaplan–Meier survival analysis of *KRAS* mutations combined with risk score. (**J**) Correlations between risk score and sensitivity to six drugs (pazopanib, phenformin, ruxolitinib, lapatinib, epothilone, and paclitaxel). Differences in drug sensitivity between the high- and low-risk groups were also analyzed.

We analyzed the TMB landscape of the high and low groups and identified the top 20 mutated genes (Fig. 9A,B). Survival rate was significantly lower in the high TMB group than in the low TMB group (P = 0.008, Fig. 9C). Joint analysis of TMB and risk score revealed that the low TMB and low-risk groups had better prognosis than the other three groups (P < 0.001, Fig. 9D). The high-risk group had higher TMB than the low-risk group (P = 0.0005, Fig. 9E). Unsurprisingly, risk score and mutation burden were positively correlated (P < 0.001, Fig. 9F). *KRAS* had the highest mutation frequency among all genes, and patients with mutated *KRAS* had higher risk scores than those with wild-type *KRAS* (P < 0.001, Fig. 9G). Moreover, survival analysis showed that patients with mutant KRAS had a worse prognosis than those with wild-type KRAS (P < 0.001, Fig. 9H). Survival analysis of the *RAS* mutation combined with risk scores showed that high-risk patients with *KRAS* mutation had a worse prognosis than low-risk patients with wild-type *KRAS* (P < 0.001, Fig. 9I). Drug sensitivity analysis showed that low-risk groups were more sensitive to pazopanib, phenformin, and ruxolitinib; furthermore, sensitivity was negatively correlated with risk score (P < 0.05). In contrast, high-risk groups were more sensitive to lapatinib, epothilone, and paclitaxel; sensitivity was positively correlated with the risk score (P < 0.05, Fig. 9J).

## Discussion

RNA methylation regulates cancer development and progression through its effects on RNA metabolism^33^. Recent research has focused on the methylation of lncRNAs and their involvement in cancers. For instance, in pancreatic cancer, IGF2BP2 acts as an m6A reader to regulate the expression of DANCR, thus promoting the proliferation and invasion of pancreatic cancer cells^34^. NSUN2 functions as a coder (writer) of m5C in the development and pathogenesis of many cancers (e.g., breast, lung, and colorectal cancers)^35^. ALKBH3 acts as an eliminator (eraser) for m1A, thereby regulating NADH oxidase and Fn14/VEGF signaling to promote the development and progression of human uroepithelial carcinoma^36^. Our findings increase our current understanding on the involvement of RNA methylation-related lncRNAs in cancer. We identified reliable lncRNA diagnostic markers for patients with early pancreatic cancer. These biomarkers can also help assess prognosis, response to immunotherapy, and effective drugs, all of which should benefit the development of individualized treatment plans for patients with pancreatic cancer.

Analysis of survival data and the expression profiles of 177 pancreatic cancer samples uncovered 218 lncRNAs that are highly correlated with methylation. Among these, 39 lncRNAs were related to both prognosis and methylation, with 15 being deleterious (HR > 1) and the remaining 24 being protective (HR < 1). As we expected, their expression differed significantly between tumor and non-tumor samples. Next, we clustered the lncRNAs based on their expression profiles and found that Cluster1 was associated with significantly longer survival than Cluster2. The cause of this difference was revealed by comparing the immune microenvironment of the two subgroups. We found that Cluster1 was associated with significantly higher tumor purity, immune score, stromal score, and ESTIMATE score than Cluster2, thereby confirming our suspicion that immunity plays a major role in prognosis.

We obtained a model including 39 lncRNAs. To reduce model complexity while enhancing stability and usability, we further screened out seven lncRNAs (SOCS2-AS1, LINC00941, UCA1, CASC8, TM4SF1-AS1, SNHG10, and DNMBP-AS1). SOCS2-AS1 is a lncRNA transcribed from the antisense strand of *SOCS2*^37^ and is associated with tumor development, being aberrantly expressed in colorectal cancer, prostate cancer, renal clear cell carcinoma, and glioma. For example, circ-ASPH overexpression promoted the proliferation of glioma and prostate cancer cells, subsequently promoting progression by positively regulating the miR-599/AR/SOCS2-AS1 axis^38^. Moreover, SOCS2-AS1 overexpression in colorectal cancer inhibited the miR-1264-mediated positive regulation of *SOCS2*. Both in vitro and in vivo results demonstrated that this overexpression inhibited colorectal cancer cell proliferation, migration, and invasion^39^. To our knowledge, this is the first study to report the association between *SOCS2* and pancreatic cancer. Univariate regression and differential expression analyses revealed that SOCS2-AS1 expression was decreased in the high-risk group and increased in the low-risk group. This finding suggests that SOCS2-AS1 may be a protective factor for pancreatic cancer.

Prior in vivo and ex vivo experiments, in combination with bioinformatics analysis, revealed that high LINC00941 expression contributes to poor prognosis in pancreatic cancer patients. The mechanism of action may involve the mediation of metabolic reprogramming in pancreatic cancer cells, activation of the Hippo pathway, and enhancement of glycolysis in PDAC^40^. Additionally, LINC00941 may promote the proliferation of pancreatic cancer cells, leading to metastasis through competitive binding with miR-873-3p^41^.

UCA1 (located on chromosome 19p13.12) was first reported in bladder cancer and is aberrantly expressed in multiple tumors^42^. In depleted cells, UCA1 acts as an exosomal lncRNA to promote angiogenesis in pancreatic cancer via the miR-96-5p/AMOTL2 axis. It may also be involved in the development of cancer drug resistance^43,44^.

CASC8 is located on chromosome 8q24, and its specific upregulation in pancreatic adenocarcinoma might indicate poor prognosis^45^. These findings are consistent with our results. Furthermore, its association with poor prognosis may be attributed to its interaction with H19, miR-671, and SMAD7. CASC8 might be involved in epithelial mesenchymal transition (EMT) and pancreatic adenocarcinoma progression.

High TM4SF1-AS1 expression resulted in the activation of the TM4SF1 and PI3K-AKT pathways in gastric cancer cells, thereby enhancing proliferation, invasion, and EMT^46^. Subsequently, TM4SF1 promoted pancreatic cancer metastasis by regulating DDR1 expression^47^. These previous findings support our result, i.e., high TM4SF1-AS1 expression in patients with pancreatic cancer is linked to poor prognosis.

Most studies on SNHG10 and DNMBP-AS1 have been conducted in cancer types other than pancreatic cancer. For instance, SNHG10 promotes the growth and migration of gastric tumors by targeting the miR-495-3p/CTNNB1 axis, activating the WNT pathway and the DDX54-mediated PBX3 feedback pathway^48,49^. SNHG10 also plays an important role in the progression of prostate cancer, lung cancer, and osteosarcoma. Finally, DNMBP-AS1 is an miRNA sponge that competitively binds to miR-93-5p/17-5p to promote NHLRC3 expression, thus inhibiting colon cancer progression^50^.

To explore the diagnostic and therapeutic value of the hub lncRNAs, we performed separate correlation analyses for each lncRNA and risk score. We then performed survival analysis of tumor samples based on their expression. The results indicated that CASC8, DNMBP-AS1, LINC00941, TM4SF1-AS1, and UCA1 were positively correlated with the risk scores, while the high-expression group had significantly better prognosis than the low-expression group. The opposite was true for SNHG10 and SOCS2-AS1. Thus, these seven lncRNAs have independent diagnostic value as promising biomarker candidates and valuable therapeutic targets for pancreatic cancer.

The risk scores calculated for each sample were positively correlated with mortality rates, and their independent prognostic power was verified using COX regression. Furthermore, we constructed a nomogram by combining clinical characteristics and risk scores. Results from the c-index and calibration curves indicate that the combined model has a high validity and reliability.

Immunotherapy is increasingly valued as a cancer treatment because it has substantial therapeutic effects with a few side effects. The immunotherapy drugs currently available for clinical practice include immune-checkpoint inhibitors, adoptive cell transfer therapy, tumor-specific vaccines, and small-molecule immune drugs^51^. In particular, immune-checkpoint inhibitors are research hotspots as they ensure rapid and lasting therapeutic effects for cancer patients. Unfortunately, pancreatic ductal adenocarcinoma is resistant to immunotherapy, and immune-checkpoint inhibitors have limited effects^52,53^. Therefore, physicians require a method for assessing the response to immunotherapy, such that they can select patients who are more responsive to such treatments. Here, we addressed this issue through an in-depth analysis of our model’s immune landscape. The results of immune microenvironment analysis were consistent with the correlations between immune cells and risk scores. Activated dendritic cells, M0 macrophages, and resting memory CD3 T cells were all positively correlated with the risk scores, whereas CD8 T cells and monocytes were negatively correlated with the risk scores. As we expected, tumor immune infiltration scores were in alignment with the subgroup patterns. We then used TIDE to determine which subgroup is more likely to benefit from immunotherapy. The TIDE computational model predicts the therapeutic effect of immune-checkpoint inhibitors on different subgroups by simulating the two main mechanisms of tumor immune evasion. Our TIDE scores indicated that immunotherapy will be more helpful for the high-risk group. We also found that elevated expression of TM4SF1-AS1, CASC8, UCA1, and LINC00941 was associated with C1, C2, and C6, suggesting a tumor-promoting effect and poor prognosis.

Another method of predicting the response to immunotherapy is through TMB^54^. This emerging biomarker correlated with patient survival^55^. In this study, we observed that the high-risk group had a significantly higher TMB than the low-risk group. Additionally, survival time was significantly shorter in the high-TMB group than in the low-TMB group. Furthermore, *KRAS* was the most frequently mutated gene, with an 83% mutation rate in the high-risk group and a 37% mutation rate in the low-risk group. This outcome corroborates that of previous studies showing that *KRAS* is among the most commonly mutated genes in pancreatic cancer, with a mutation frequency of approximately to 100% in pancreatic ductal adenocarcinoma^56^, and that it is closely associated with poor prognosis^57^. Moreover, a study on KPC mice reported that *KRAS* (G12D) and Trp53 (R172H) promoted pancreatic cancer development and metastasis^58^. In our study, patients with mutant *KRAS* had higher risk scores and shorter survival than those with wild-type *KRAS*. As expected, the strategy of combining *KRAS* mutation and risk scores revealed that high-risk patients with mutated *KRAS* had the worst prognosis, whereas low-risk patients with wild-type *KRAS* had the best prognosis. Overall, these data suggest that *KRAS* has considerable utility in the prognostic assessment and treatment of pancreatic cancer.

Finally, drug sensitivity analysis identified six drugs. Epothilones and paclitaxel are antitumor compounds that can inhibit microtubule depolymerization^59,60^. Pazopanib and lapatinib are both tyrosine-kinase inhibitors. However, the former has a broader range of targets, including vascular endothelial growth factor receptors (VEGFRs) 1, 2, and 3; platelet-derived growth factor receptors (PDGFR) α and β, etc.^61^. Given its ability to inhibit numerous targets, pazopanib can potentially be used to treat pancreatic cancer^62^. Phenformin was originally discovered as a hypoglycemic drug but has been withdrawn, as it greatly increases the risk of lactic acidosis. Nonetheless, continued research on phenformin has revealed remarkable and unique antitumor effects^63^. Finally, ruxolitinib is an inhibitor of the JAK signaling pathway and by extension, the inflammatory signaling pathway downstream of IL-6R. Notably, the systemic inflammatory response in patients with pancreatic cancer is very closely associated with a poorer prognosis^64^. This factor, combined with our results, suggests that this drug may have unexpected benefits for patients with pancreatic cancer; however, further experimental studies are needed for validation.

Our current study had some limitations. First, our model of methylation-associated lncRNAs lacked external validation. Although model lncRNAs have been investigated in other cancers, the relevant molecular mechanisms should be investigated to confirm how the lncRNAs we identified are involved in pancreatic cancer.

Circulating lncRNAs are recognized as reliable diagnostic and prognostic biomarkers and potential therapeutic targets^65^. Hence, the lncRNAs we identified may be valuable in the diagnosis of early pancreatic cancer. In future research, we aim to further explore their mechanism of action in pancreatic cancer.

## Conclusions

Our findings highlighted seven methylation-related lncRNAs associated with pancreatic cancer prognosis and have potential as biomarkers. Our study also demonstrated that methylation-related lncRNAs are strongly correlated with the immune microenvironment, mutational burden, and response to immunotherapy in pancreatic cancer. These findings will greatly benefit the efforts to develop multidisciplinary treatment models and individualized therapy.

## Supplementary Information

Supplementary Information.

## Data Availability

All data generated or analyzed during this study are included in the published article and its supplementary information files.
