# Supplementary material for: Identification of m6A/m5C/m1A-associated LncRNAs for prognostic assessment and immunotherapy in pancreatic cancer
Source: Sci Rep. 2023 Mar 4;13:3661. doi: 10.1038/s41598-023-30865-9 (PMC9985641; doi:10.1038/s41598-023-30865-9)
Supplement: Supplementary file 1 — Supplementary Information. [file 41598_2023_30865_MOESM1_ESM.zip › Supplementary Figure S4.pdf]

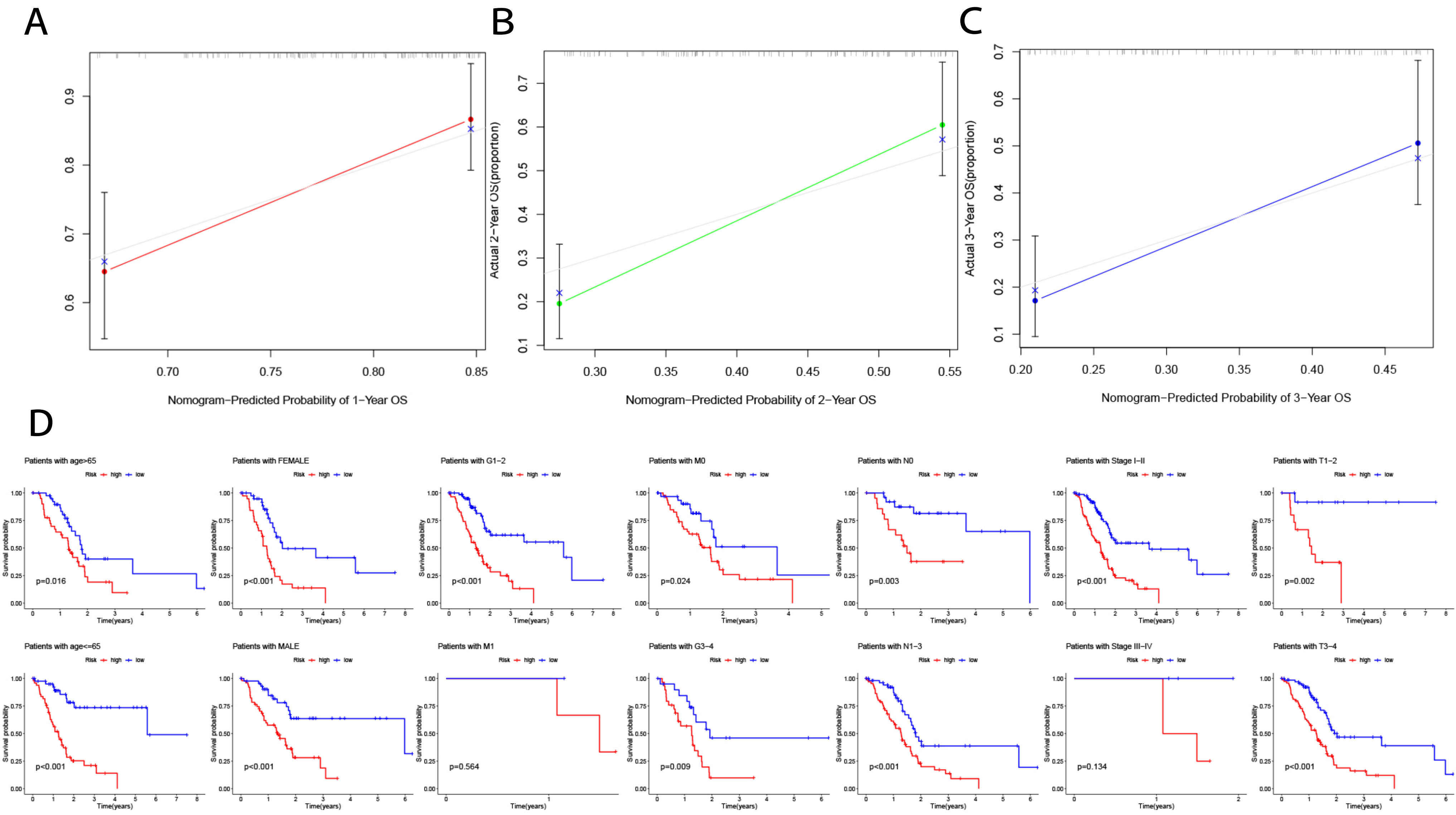
A
B
C
D
Nomogram−Predicted Probability of 1−Year OS
Nomogram−Predicted Probability of 2−Year OS
Nomogram−Predicted Probability of 3−Year OS
Actual 2−Year OS(proportion)
Actual 3−Year OS(proportion)
Patients with age>65
Patients with FEMALE
Patients with G1−2
Patients with M0
Patients with N0
Patients with Stage I−II
Patients with T1−2
Patients with age<=65
Patients with MALE
Patients with M1
Patients with G3−4
Patients with N1−3
Patients with Stage III−IV
Patients with T3−4
Risk high low
Survival probability
Time(years)
p=0.016
p<0.001
p<0.001
p=0.024
p=0.003
p<0.001
p=0.002
p<0.001
p<0.001
p=0.564
p=0.009
p<0.001
p=0.134
p<0.001
