# Supplementary material for: Identification of m6A/m5C/m1A-associated LncRNAs for prognostic assessment and immunotherapy in pancreatic cancer
Source: Sci Rep. 2023 Mar 4;13:3661. doi: 10.1038/s41598-023-30865-9 (PMC9985641; doi:10.1038/s41598-023-30865-9)
Supplement: Supplementary file 1 — Supplementary Information. [file 41598_2023_30865_MOESM1_ESM.zip › Supplementary legends.doc]

**Supplementary Figure S1.** Heatmap of clinical parameters in Cluster1 and Cluster2.

**Supplementary Figure S2.** Correlations between KRAS genes and prognostic lncRNAs.

**Supplementary Figure S3.** Diagnostic efficacy of risk models. (**A-C**) ROC values for risk scores and clinical parameters at 1, 2, and 3 years. (**D**) Conformance index of risk score and clinical parameters.

**Supplementary Figure S4.** Prognostic value of risk models. (**A–C**) Calibration curves for 1, 2, and 3 years post-diagnosis. (**D**) Survival analysis of clinical parameters between high- and low-risk groups.

**Supplementary Figure S5.** The value of model lncRNAs. (**A**) Survival analysis of model lncRNAs. (**B**) Correlations between model lncRNAs and risk scores.

**Supplementary Table 1.** Clinicopathological characteristics of patients with pancreatic ductal adenocarcinoma in TCGA.
