# Supplementary material for: Identification of m6A/m5C/m1A-associated LncRNAs for prognostic assessment and immunotherapy in pancreatic cancer
Source: Sci Rep. 2023 Mar 4;13:3661. doi: 10.1038/s41598-023-30865-9 (PMC9985641; doi:10.1038/s41598-023-30865-9)
Supplement: Supplementary file 1 — Supplementary Information. [file 41598_2023_30865_MOESM1_ESM.zip › Supplementary Table 1.docx]

Table 1: Clinicopathological characteristics of patients with pancreatic ductal adenocarcinoma in TCGA.

|  | Type | Text(N=88) | Train(N=89) | Total(N=177) | Pvalue |
| --- | --- | --- | --- | --- | --- |
| staus | death | 45(25.42%) | 47(26.55%) | 92(51.98%) | 0.94 |
| staus | alive | 43(24.29%) | 42(23.73%) | 85(48.02%) |  |
| age | <=65 | 44(24.86%) | 49(27.68%) | 93(52.54%) | 0.6 |
| age | >65 | 44(24.86%) | 40(22.60%) | 84(47.46%) |  |
| gender | female | 44(24.86%) | 36(20.34%) | 80(45.20%) | 0.26 |
| gender | male | 44(24.86%) | 53(29.94%) | 97(54.80%) |  |
| grade | G1-2 | 62(35.03%) | 63(35.59%) | 125(70.62%) | 0.35 |
| grade | G3-4 | 24(13.56%) | 26(14.69%) | 50(28.25%) |  |
| grade | GX | 2(1.13%) | 0(0.0e+0%) | 2(1.13%) |  |
| stage | Stage I-II | 83(46.89%) | 84(47.46%) | 167(94.35%) | 0.12 |
| stage | Stage III-IV | 5(2.82%) | 2(1.13%) | 7(3.95%) |  |
| stage | Stage X | 0(0.0e+0%) | 3(1.69%) | 3(1.69%) |  |
| T | T1-2 | 18(10.17%) | 13(7.34%) | 31(17.51%) | 0.23 |
| T | T3-4 | 70(39.55%) | 74(41.81%) | 144(81.36%) |  |
| T | TX | 0(0.0e+0%) | 2(1.13%) | 2(1.13%) |  |
| M | M0 | 36(20.34%) | 43(24.29%) | 79(44.63%) | 0.41 |
| M | M1 | 3(1.69%) | 1(0.56%) | 4(2.26%) |  |
| M | MX | 49(27.68%) | 45(25.42%) | 94(53.11%) |  |
| N | N0 | 25(14.12%) | 24(13.56%) | 49(27.68%) | 0.89 |
| N | N1 | 61(34.46%) | 62(35.03%) | 123(69.49%) |  |
| N | NX | 2(1.13%) | 3(1.69%) | 5(2.82%) |  |
