# Supplementary figures and images for: Identification of m6A/m5C/m1A-associated LncRNAs for prognostic assessment and immunotherapy in pancreatic cancer
Source: Sci Rep. 2023 Mar 4;13:3661. doi: 10.1038/s41598-023-30865-9 (PMC9985641; doi:10.1038/s41598-023-30865-9)

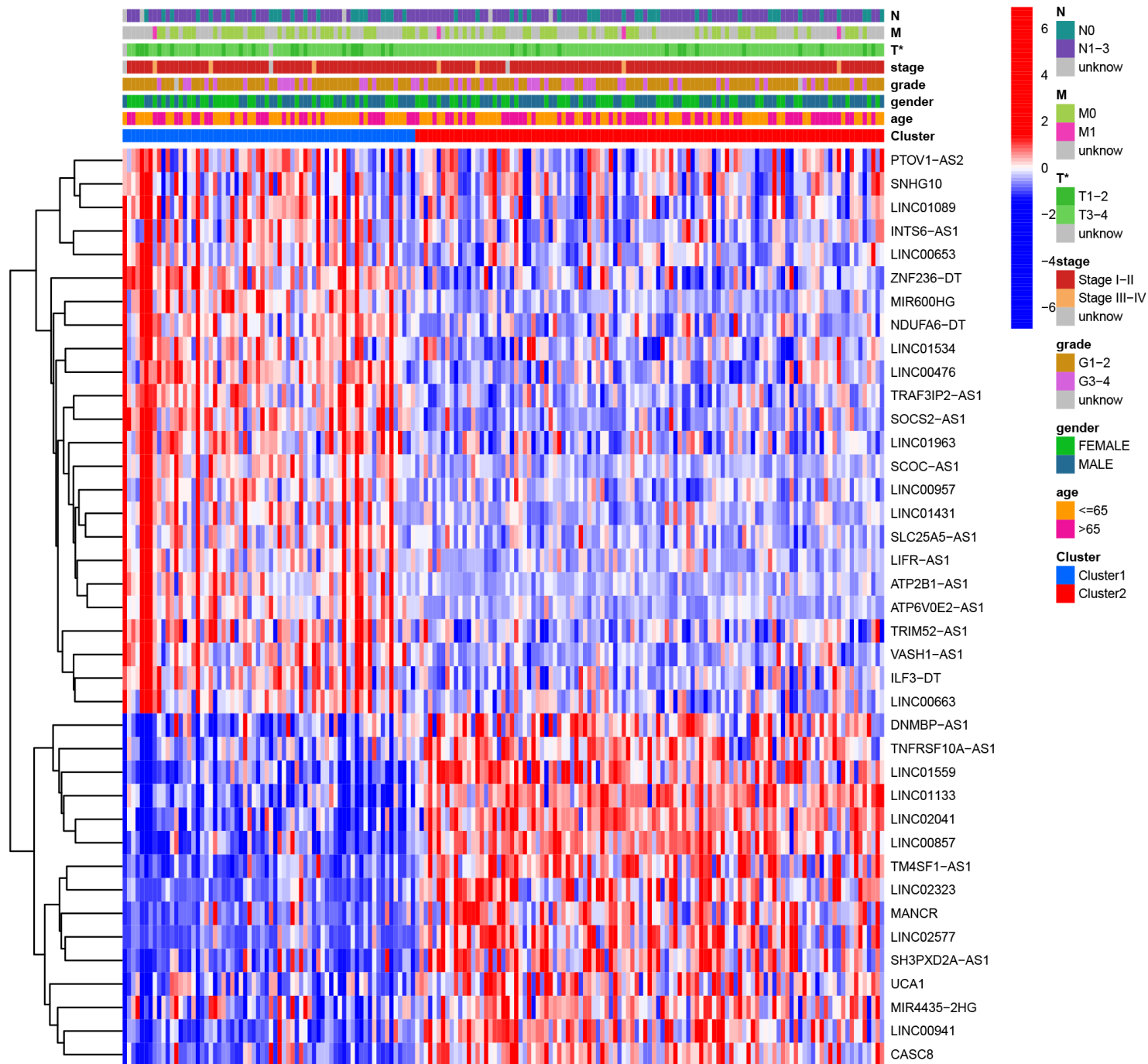

Supplement: Supplementary file 1 — Supplementary Information. [file 41598_2023_30865_MOESM1_ESM.zip › Supplementary Figure S1.pdf]

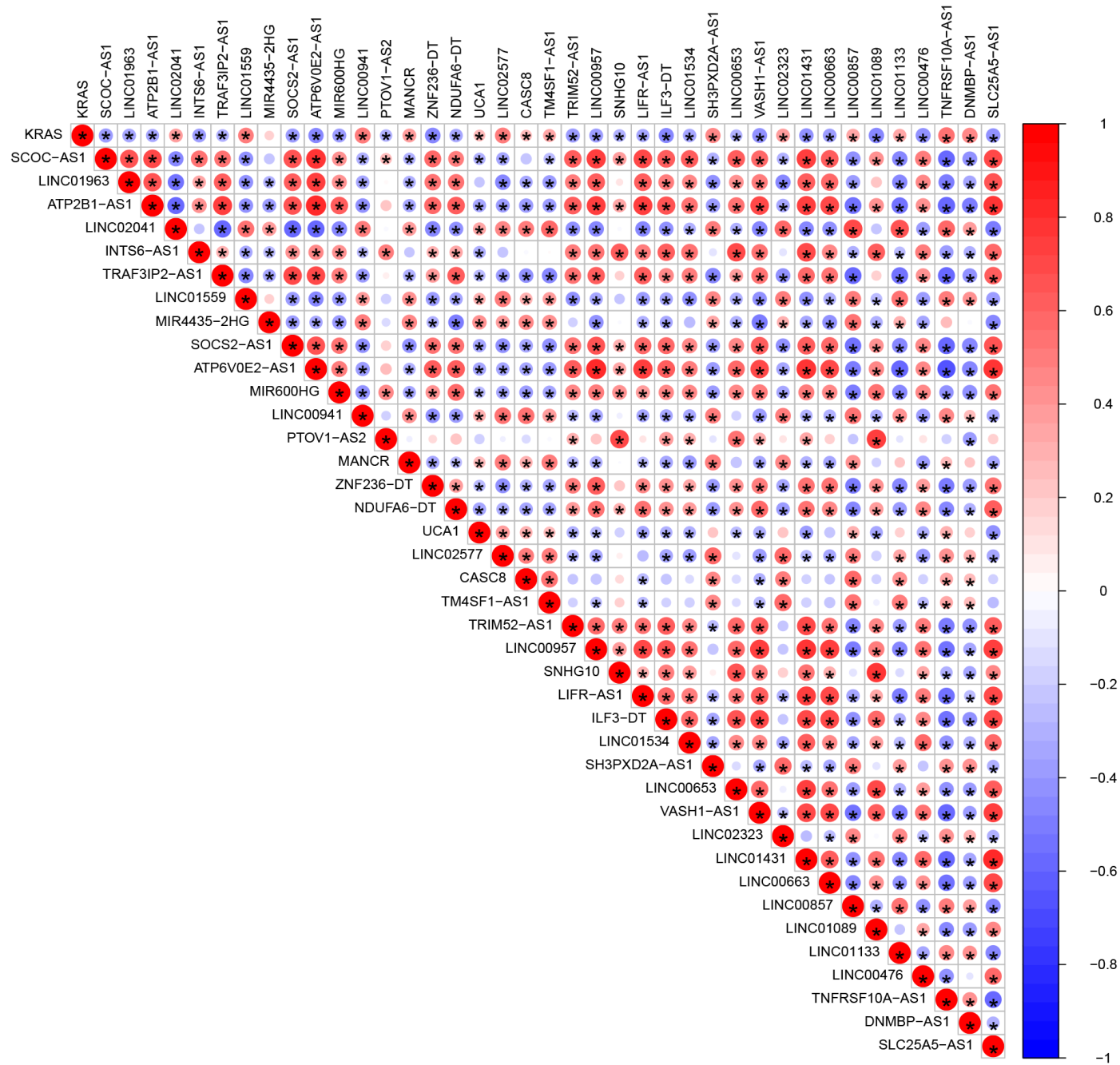

Supplement: Supplementary file 1 — Supplementary Information. [file 41598_2023_30865_MOESM1_ESM.zip › Supplementary Figure S2.pdf]

**A**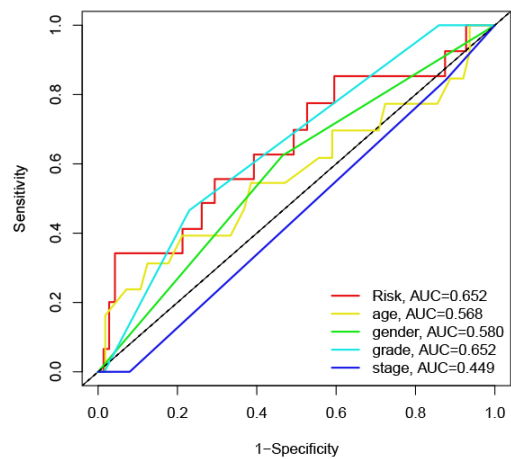**B**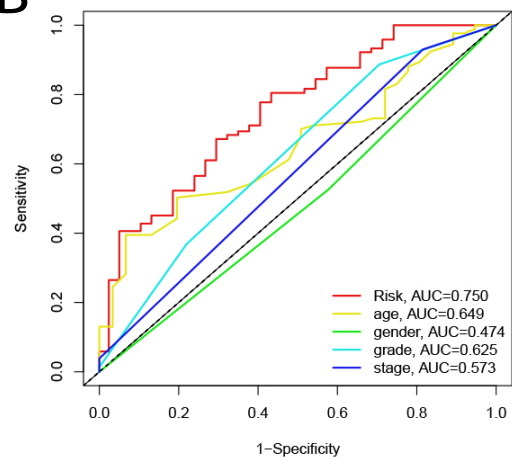**C**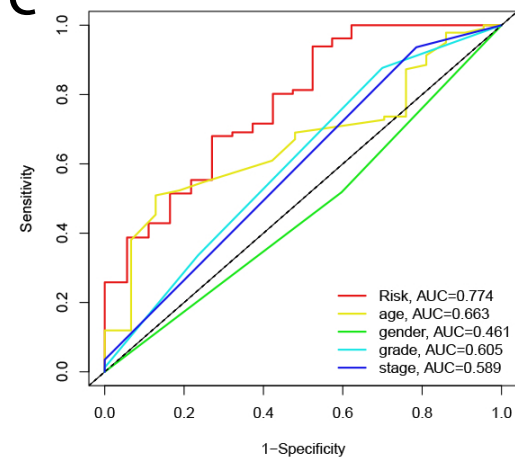**D**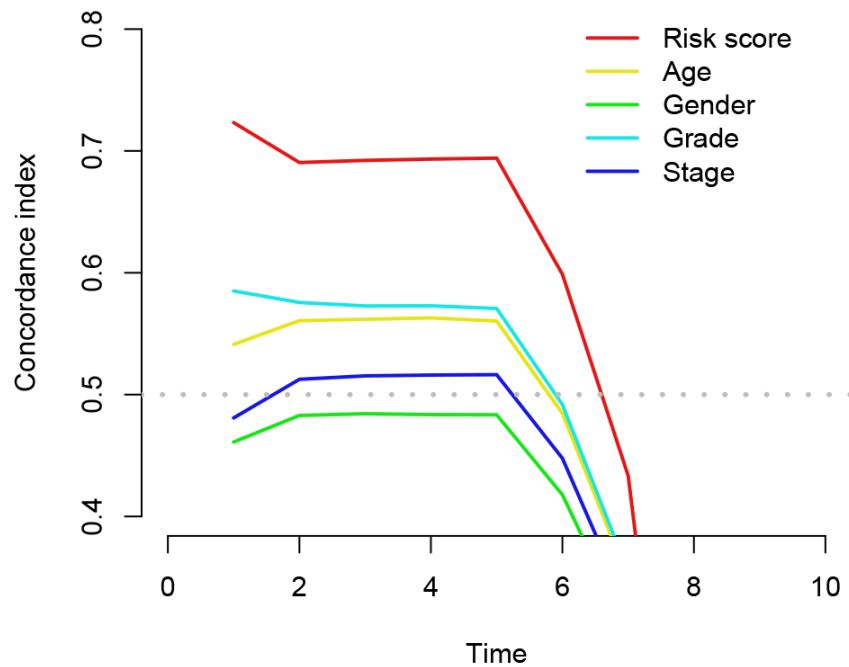

Supplement: Supplementary file 1 — Supplementary Information. [file 41598_2023_30865_MOESM1_ESM.zip › Supplementary Figure S3.pdf]

A

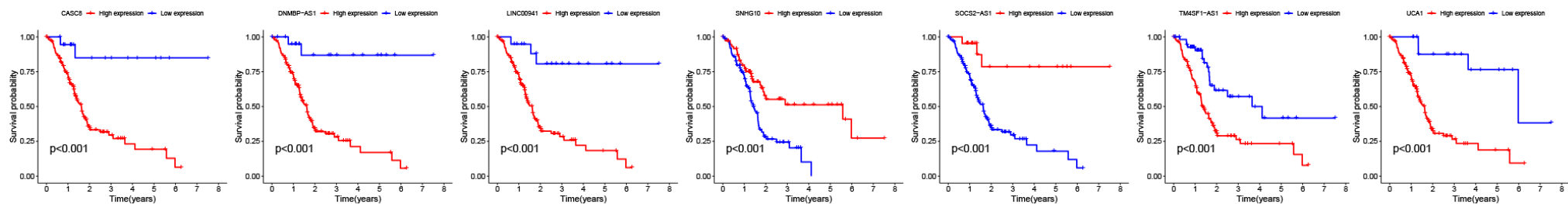

B

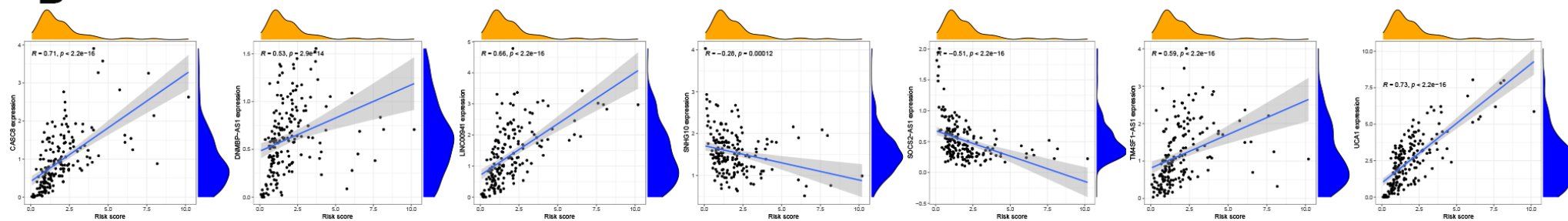

Supplement: Supplementary file 1 — Supplementary Information. [file 41598_2023_30865_MOESM1_ESM.zip › Supplementary Figure S5.pdf]
